# Supplementary material for: Tetra-2,3-Pyrazinoporphyrazines with Externally Appended Pyridine Rings 22 Synthesis, Physicochemical and Photoactivity Studies on In(III) Mono- and Heteropentanuclear Complexes
Source: Molecules. 2022 Jan 27;27(3):849. doi: 10.3390/molecules27030849 (PMC8840402; doi:10.3390/molecules27030849)
Supplement: Supplementary file 1 [file molecules-27-00849-s001.zip › molecules-1543235-supplementary.pdf]

Tetra-2,3-pyrazinoporphyrazines with Externally Appended Pyridine Rings 22 Synthesis, Physicochemical and Photoactivity Studies on In(III) Mono- and Heteropentanuclear Complexes.

Maria Pia Donzello,[a] Giulia Capobianco,[a] Ida Pettiti,[a] Claudio Ercolani,[a] Pavel A. Stuzhin[b]

[a] *Dipartimento di Chimica, Università di Roma Sapienza, P. le A. Moro 5, I-00185 Rome, Italy*
[b] *Department of Organic Chemistry, Ivanovo State University of Chemistry and Technology, 153460 Ivanovo, Russian Federation*

Supporting Information

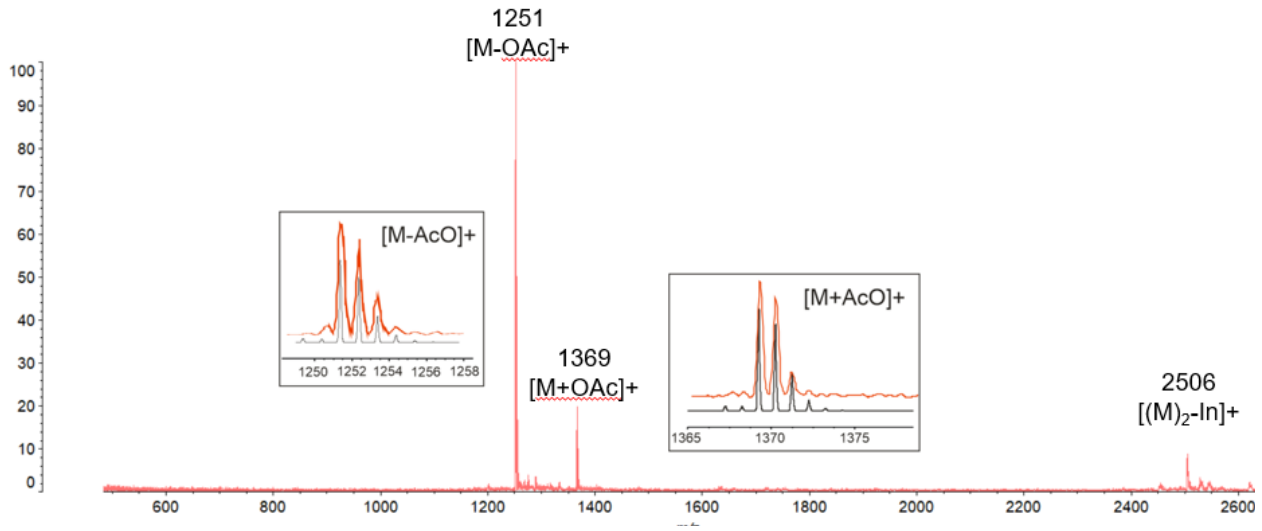

Figure S1. Mass spectrum of the complex [Py_8TPyzPzIn(OAc)]·8H_2O from solution in MeCN. Insets shown the isotopic distribution pattern.

Comments to Figure S1

In the MALDI mass-spectrum of [Py_8TPyzPzInOAc]·8H_2O recorded from a solution in CH_3CN the ion axially carrying the In-Cl group [Py_8TPyzPzInCl]$^+$ at 1287.5 Da is not present and the molecular ion of the acetate complex [M]+ at 1310 Da is also not seen; instead, the intense peak is present at 1251 Da, accompanied by minor peaks at 1369 Da (20%) and 2506 Da (10%). The peak at 1251 Da corresponds to the stable fragmentation cation [M-OAc]+ formed by loss of the coordinated acetate anionand the peak at 1369 Da corresponds to the association ion [M+OAc]$^+$. It is known from the reported literature that phthalocyanine-In^{III} complexes coordinate carboxylate type anions in a bidentate fashion[1] and coordination number of In^{III} allows attachment of two such ions in cis-position.[2] In the complexes with bidentate coordinated carboxylates the In^{III} atom is located ca 0.9 Å out of the macrocyclic plane, which is more than in the case of monodentate complexes (ca 0.9 Å). Coordination of two bidentate ions leads to further external positioning of the metal from the macrocyclic plane (>1.05 Å) in the case of bis(nitrito) complexes and this additional coordination facilitates the demetallation.[3,4] In addition, In(III) can form stable sandwich complexes with the phthalocyanine type macrocycles.[5] In view of this, appearance of the peaks at 2506 Da corresponding to the dimeric ion [$(M)_2$-In]+ with loss of one In atom is well understood. It was reported that In^{III} phthalocyaninates form easily oligomeric species with bridging acetate ligands.[6]

References

1) K Schweiger, K.; Kienast, A.; Latte, B; H. Homborg. Darstellung und Eigenschaften von Phthalocyaninato(2−)indaten(III) mit zweizähnigen Oxo-Liganden; Kristallstruktur von Tetra(n-butyl)ammoniumcarbonato(*O,O′*)-phthalocyaninato(2−)indat(III). *Z. anorg. allg. Chem.* **1997**, *623*, 973-980

2) Aßmann, B.; ,Ostendorp, G.; Homborg, H.n^{III}-Phthalocyanine: Darstellung, Eigenschaften und Kristallstruktur von Tetra(*n*-butyl)ammonium-cis-di(nitrito-*O,O′*)phthalocyaninato(2–) indat(III). *Z. anorg. allg. Chem.* **1997**, *621*, 1708-1714.

3) Svetlana, S. I.; Stuzhin, P. A. Unusual catalytic effect of halide anions in the protolytic dissociation of indium(III) octaphenyltetraazaporphyrins. *Mendeleev Commun.s* **2004**, 229-320.

4) Svetlana, S. I.; Sthuzhin, P. A. Indium(III) complexes of octaphenylporphyrazine: Effect of halide coordination on the basic properties and stability in acid media. *J. Porphy. Phthalocyanines* **2011**, 15, 1299-1309.

5) Ostendorp, G.; Homborg, H. Darstellung und Eigenschaften der Diphthalocyaninate des Yttriums und Indiums *Z. anorg. allg. Chem.* **1996**, *622*, 1358-1364.

6) Janczak, J.; Kubiak, R. From iodoindium(III) phthalocyanine to the π-radical indium(III) diphthalocyanine and magnetically frustrated indium diacetate hydroxide coordination polymer. *Inorg. Chim. Acta* **2011**, *376*, 28–35.

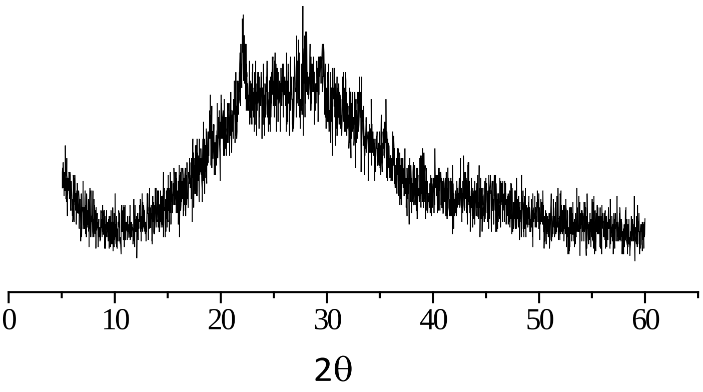

Figure S2. X-ray powder spectrum of the complex $[(2\text{-Mepy})_8TPyzPzIn(OAc)](I)_8 \cdot H_2O$.

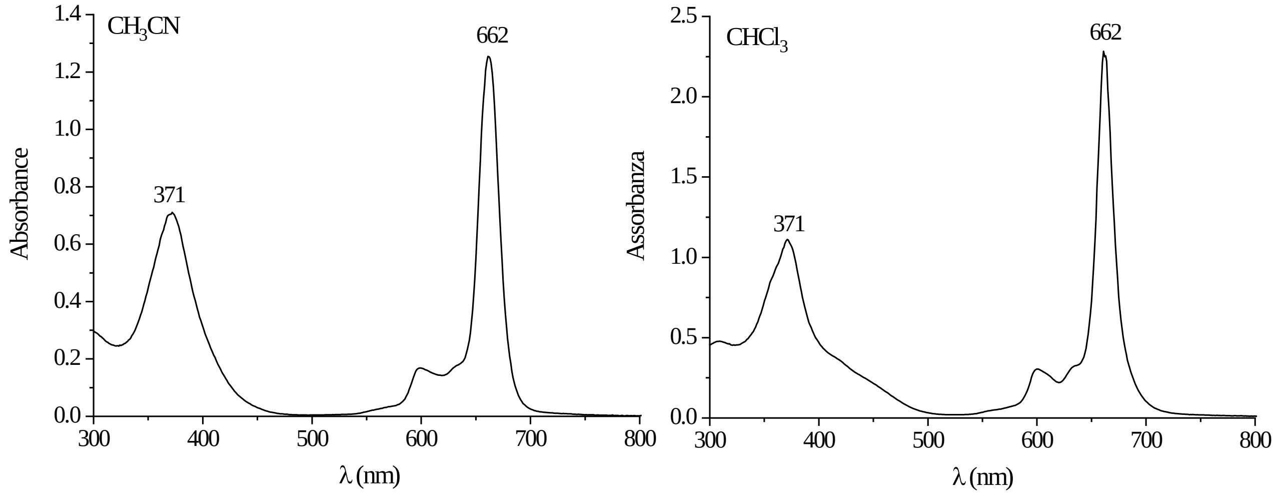

Figure S3. UV-visible spectra in the range 300-800 nm of $[Py_8TPyzPzIn(OAc)]\cdot 8H_2O$ in CH_3CN (left) and $CHCl_3$ (right).

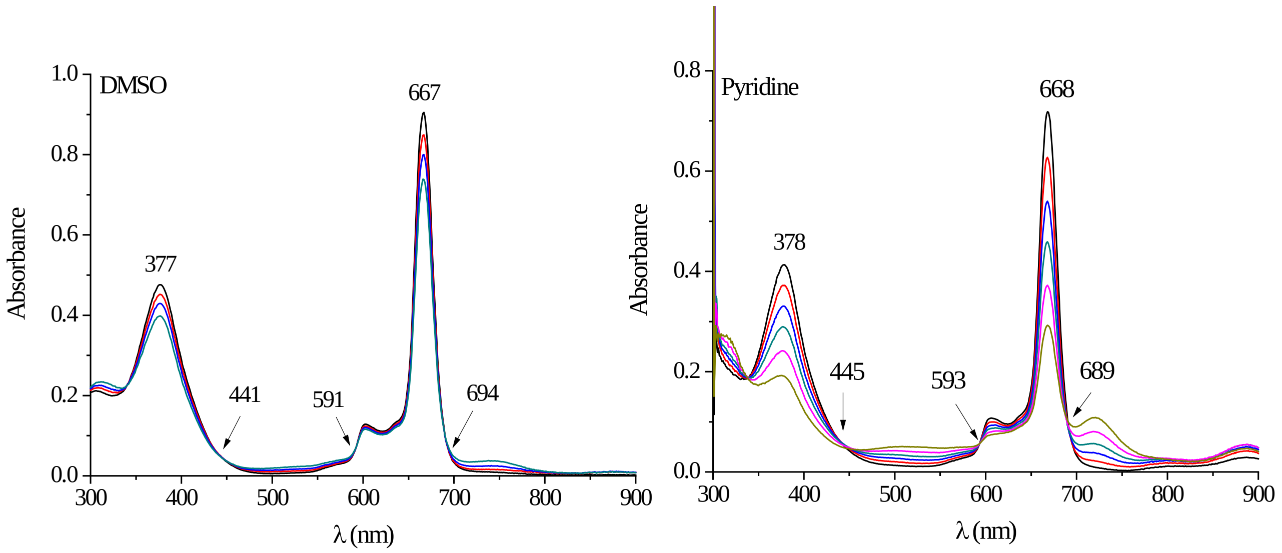

Figure S4. UV-visible spectra in the range 300-900 nm of $[Py_8TPyzPzIn(OAc)]\cdot 8H_2O$ in DMSO (left) and pyridine (right).

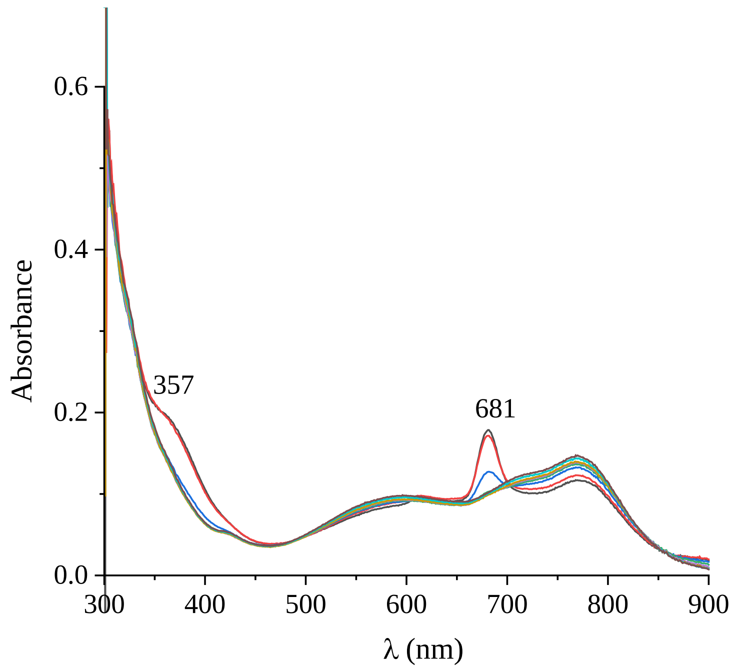

Figure S5. UV-visible spectra in the range 300-900 nm of the complex $[(2\text{-Mepy})_8\text{TPyzPzIn(OAc)}](\text{I})_8{\cdot}\text{H}_2\text{O}$ in pyridine.

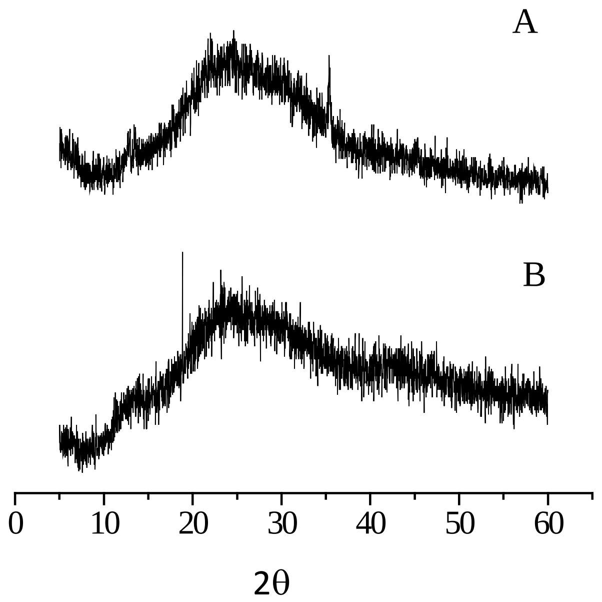

Figure S6. X-ray powder spectra of the pentanuclear complexes $[(\text{PdCl}_2)_4\text{Py}_8\text{TPyzPzIn(OAc)}]{\cdot}8\text{H}_2\text{O}$ (A), $[(\text{PtCl}_2)_4\text{Py}_8\text{TPyzPzIn(OAc)}]{\cdot}\text{H}_2\text{O}$ (B).

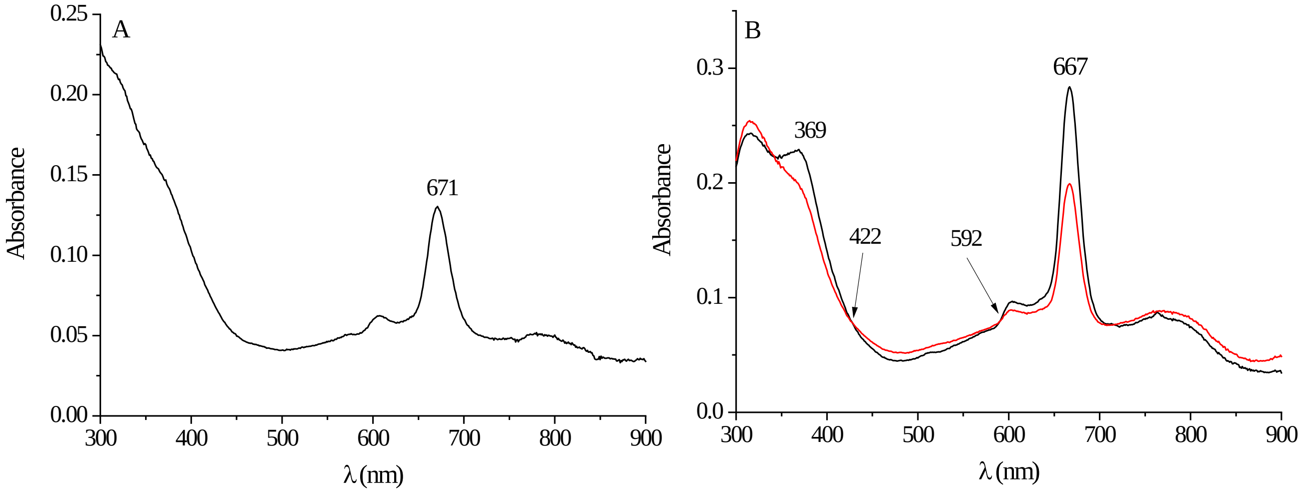

Figure S7. UV-visible spectra in the range 300-900 nm of the pentanuclear complexes $[(PdCl_2)_4Py_8TPyzPzIn(OAc)]\cdot 8H_2O$ (A) and $[(PtCl_2)_4Py_8TPyzPzIn(OAc)]\cdot H_2O$ (B) in DMF.

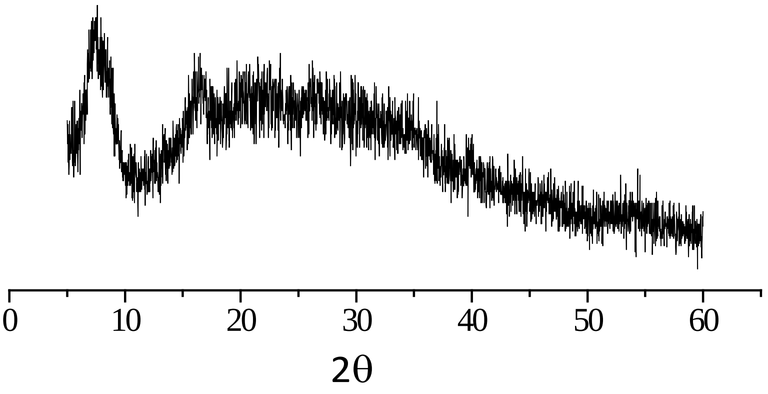

Figure S8. X-ray powder spectrum of the complex $[\{Pd(CBT)_2\}_4Py_8TPyzPzIn(OAc)]\cdot 19H_2O$
